# Expression of microRNA-150 and its Target Gene IGF2BP1 in Human Osteosarcoma and their Clinical Implications

**DOI:** 10.1007/s12253-018-0454-0

**Published:** 2018-09-15

**Authors:** Lei Wang, Ailixiati Aireti, Aizezi Aihaiti, Kun Li

**Affiliations:** Department of Orthopedics Center Spine Surgery, People’s Hospital of Xinjiang Uygur Autonomous Region, Urumqi, 830000 China

**Keywords:** Osteosarcoma, microRNA-150, Insulin-like growth factor 2 mRNA-binding protein 1, Clinicopathological feature, Overall survival, Disease-free survival

## Abstract

Previous study revealed that microRNA (miR)-150 might function as a tumor suppressor in osteosarcoma partially by targeting Insulin-Like Growth Factor 2 mRNA-Binding Protein 1 (IGF2BP1). The aim of this study was to investigate the clinical significance of miR-150-IGF2BP1 axis in human osteosarcoma which remains unclear. At first, expression levels of miR-150, and IGF2BP1 mRNA and protein in 20 osteosarcoma and matched adjacent noncancerous tissues were respectively detected by quantitative real-time PCR and western blot analyses. Then, subcellular localization and expression pattern of IGF2BP1 protein in 100 osteosarcoma tissues were examined by immunohistochemistry. Associations of miR-150/IGF2BP1 expression with various clinicopathological features and patients’ prognosis were also statistically evaluated. As a result, miR-150 expression was significantly decreased, while IGF2BP1 mRNA and protein expression were dramatically increased in osteosarcoma tissues compared to matched adjacent noncancerous tissues (all *P* < 0.001). Immunostaining of IGF2BP1 protein was localized in cytoplasm of tumor cells in osteosarcoma tissues. Statistically, low miR-150 expression and/or high IGF2BP1 protein immunoreactive score were all significantly associated with high tumor grade, presence of metastasis and recurrence, as well as poor response to chemotherapy (all *P* < 0.05). Moreover, miR-150, IGF2BP1 and combined miR-150/IGF2BP1 expressions were all identified as independent prognostic factors for overall and disease-free survivals of osteosarcoma patients (all *P* < 0.05). In conclusion, our data suggest that miR-150 and its downstream target IGF2BP1 may be a crucial axis for the development, progression and patients’ prognosis of ostesarcoma. The newly identified miR-150/IGF2BP1 axis might be a novel potential therapeutic target for osteosarcoma treatment.

## Introduction

Osteosarcoma is the most common type of bone tumor among children, adolescents, and young adults worldwide, representing a leading cause of cancer-related death [1]. This malignancy is predominantly occurring around regions with active bone growth and repair, and its incidence rate is increasing [2]. Owing to the recent advances in therapeutic strategies for osteosarcoma, such as wide tumor excision, neoadjuvant and adjuvant chemotherapy, and radiotherapy, the patients’ clinical outcome and long-term survival rates have been improved distinctly [3]. However, approximately one-third of osteosarcoma patients survive for less than 5 years and the propensity of osteosarcoma cells to disseminate to the lung is the major cause of death of this tumor [4]. Therefore, it is of great clinical significance to understand the molecular basis of osteosarcoma development and metastasis, in order to discover new therapeutic targets and develop more efficient therapies.

MicroRNAs (miRNAs) with 18–25 nucleotides in length are a class of endogenously expressed small non-coding single stranded RNAs in the human body [5]. miRNAs are involved into various biological processes, including development, growth, metabolism and maturation, as well as cell proliferation, apoptosis, differentiation, invasion and metastasis, via regulating gene expression post-transcriptionally either by degradating the targeting protein-coding RNAs or by inhibiting their translation into protein [6–8]. Accumulating studies have identified aberrant miRNA expression profiles in various human tumors, and revealed their potential roles in numerous aspects of tumorigenesis and tumor progression [9, 10]. miR-150 was originally identified as a hematopoietic cell-specific miRNA which plays a critical role in normal hematopoiesis and hematological malignancies by affecting the differentiation of numerous hematopoietic cell lineages [11]. Recently, a large number of studies have observed the abnormal expression of miR-150 in various types of human tumors, and revealed that miR-150 may function as either an oncogene or a tumor suppressor depending on the action of its target genes in certain tumor types [12]. Especially in osteosarcoma, Li et al. [13] in 2015 reported that miR-150 inhibited cell proliferation, invasion, and metastasis, while stimulated cell apoptosis in osteosarcoma; Zhan et al. [14] in 2016 indicated that miR-150 upregulation could reduce osteosarcoma cell invasion and metastasis; Wang et al. [15] found that miR-150 expression level was lower in human osteosarcoma cell lines compared to the normal osteoblast cell line, which showed statistical significance (*P* < 0.01), and enforced expression of this miRNA might inhibit proliferation in human osteosarcoma cell lines; In the same year, Qu et al. [16] confirmed Insulin-Like Growth Factor 2 mRNA-Binding Protein 1 (IGF2BP1) as a direct target of miR-150, which could suppress cell proliferation, migration, and invasion, and induce apoptosis in vitro as well as suppressed tumor growth of osteosarcoma in vivo. These findings suggest that miR-150-IGF2BP1 axis may play potential roles in regulating osteosarcoma progression. However, its clinical significance in this malignancy remains unclear.

To address this problem, we firstly detected the expression levels of miR-150, and IGF2BP1 mRNA and protein in 20 osteosarcoma and matched adjacent noncancerous tissues by quantitative real-time PCR and western blot analyses, respectively. Then, the subcellular localization and expression pattern of IGF2BP1 protein were examined by immunohistochemistry using 100 osteosarcoma tissues. Associations of miR-150 and/or IGF2BP1 protein expression with various clinicopathological features and patients’ prognosis were also statistically evaluated.

## Materials and Methods

### Ethics Statement

The current study was approved by the Research Ethics Committee of the First Affiliated Hospital of Xinjiang Medical University, the Xinjiang Uygur Autonomous Region & the People’s Hospital of Xinjiang Uygur Autonomous Region, China.

### Patients and Tissue Samples

The clinical cohorts enrolled in the present study were the same as our previous study [16]. For quantitative real-time PCR and western blot analyses, 20 fresh osteosarcoma and matched adjacent noncancerous tissues were obtained from 20 patients with primary osteosarcomas; For immunohistochemistry, a total of 100 archived paraffin wax embedded osteosarcoma tissue specimens were obtained from 100 patients with primary osteosarcoma who underwent surgical operations at the First Affiliated Hospital of Xinjiang Medical University, the Xinjiang Uygur Autonomous Region & the People’s Hospital of Xinjiang Uygur Autonomous Region between 2005 and 2009. The clinicopathologic characteristics of the osteosarcoma patients were listed in Table 1. The detailed information of the two clinical cohorts used in the present study was provided in our previous publication [17].

**Table 1:** Associations of the expression of miR-150 and/or IGF2BP1 protein with various clinicopathological features of patients with osteosarcoma

Clinicopathological features	No. of cases (n, %)	miR-150-low (n, %)	P	IGF2BP1-high (n, %)	P	miR-150-low/ IGF2BP1-high (n, %)	P
Age (years)							
< 18	40 (40.00)	22 (55.00)	NS	20 (50.00)	NS	12 (30.00)	NS
≥ 18	60 (60.00)	30 (50.00)		31 (51.67)		20 (33.33)	
Sex							
Male	68 (68.00)	34 (50.00)	NS	33 (48.53)	NS	23 (33.82)	NS
Female	32 (32.00)	18 (56.25)		18 (56.25)		9 (28.13)	
Tumor site							
Femur	55 (55.00)	28 (50.91)	NS	28 (50.91)	NS	20 (36.36)	NS
Tibia	20 (20.00)	10 (50.00)		10 (50.00)		6 (30.00)	
Humeral bone	15 (15.00)	8 (53.33)		8 (53.33)		3 (20.00)	
Other	10 (10.00)	6 (60.00)		5 (50.00)		3 (30.00)	
Histologic type							
Osteoblastic	55 (55.00)	28 (50.91)	NS	28 (50.91)	NS	20 (36.36)	NS
Chondroblastic	20 (20.00)	10 (50.00)		10 (50.00)		6 (30.00)	
Fibroblastic	15 (15.00)	8 (53.33)		8 (53.33)		3 (20.00)	
Telangiectatic	10 (10.00)	6 (60.00)		5 (50.00)		3 (30.00)	
Tumor grade							
Low	15 (15.00)	5 (33.33)	0.02	4 (26.67)	0.02	2 (13.33)	0.01
High	85 (85.00)	47 (55.29)		47 (55.29)		30 (35.29)	
Metastasis							
Absent	60 (60.00)	16 (26.67)	0.001	15 (25.00)	0.001	2 (3.33)	<0.001
Present	40 (40.00)	36 (90.00)		36 (90.00)		30 (75.00)	
Recurrence							
Absent	70 (60.00)	26 (37.14)	0.001	25 (35.71)	0.001	7 (10.00)	<0.001
Present	30 (40.00)	26 (86.67)		26 (86.67)		25 (83.33)	
Response to pre-operative chemotherapy							
Good	50 (50.00)	17 (34.00)	0.01	16 (32.00)	0.01	6 (12.00)	0.001
Poor	50 (50.00)	35 (70.00)		35 (70.00)		26 (52.00)	

### RNA Extraction & Quantitative Real-Time PCR

The expression levels of miR-150 and IGF2BP1 mRNA in ostoesarcoma and adjacent noncancerous tissues were detected by quantitative real-time PCR according to the protocol of our previous study [17]. The primer sequences used in this study were listed as follows: for miR-150, Forward Primer 5'- GCT CTC CCA ACC CTT GTA CC - 3', Reverse Primer 5'- CGA GGA AGA AGA CGG AAG AAT - 3'; for U6, Forward Primer 5'- GCT TCG GCA GCA CAT ATA CTA AAA T -3', Reverse Primer 5'- CGC TTC ACG AAT TTG CGT GTC AT -3'; for IGF2BP1, Forward Primer 5'- CAG GAG ATG GTG CAG GTG TTT ATC C - 3', Reverse Primer 5'- GTT TGC CAT AGA TTC TTC CCT GAG C - 3'; for GAPDH, Forward Primer 5'- AAT CCC ATC ACC ATC TTC CA -3', Reverse Primer 5'- TGG ACT CCA CGA CGT ACT CA -3'. The amplification specificity was checked by melting curve analysis. Relative expression of miR-150 and IGF2BP1 mRNA were calculated based on the 2^-ΔΔCT^ method.

### Western Blot Analysis

The expression levels of IGF2BP1 protein in osteosarcoma and adjacent noncancerous tissues were detected by Western blot analysis according to the protocol of our previous study [17]. The following primary antibodies were used: IGF2BP1 protein (rabbit polyclonal antibody, Abcam, MA, USA; 1:150 dilution) and GAPDH (rabbit polyclonal antibody, Abcam, MA, USA; 1:150 dilution).

### Immunohistochemistry

Subcellular localization and expression pattern of IGF2BP1 protein in osteosarcoma tissues were examined by immunohistochemistry was performed according to the protocol of our previous study [17]. The following primary antibody was used: IGF2BP1 protein (rabbit polyclonal antibody, Abcam, MA, USA; 1:150 dilution).

### Evaluation of Immunostaining Results

The immunoreactive scores (IRSs) of IGF2BP1 protein were evaluated by two independent pathologists who were blinded to the clinical information of osteosarcoma patients enrolled in the current study, and calculated by integrating the proportion of positive cells and the intensity of immunoreactivity based on the description of the previous studies [18–20].

### Statistical Analysis

All statistical analyses were performed using the SPSS statistical package (version 11.0 for Windows, SPSS Inc., IL, USA). Continuous variables were expressed as mean ± S.D.. Differences of miR-150, IGF2BP1 mRNA and protein expression between osteosarcoma and the corresponding noncancerous bone tissues were evaluated by the paired *t* test. Correlation between IGF2BP1 mRNA and protein expression in osteosarcoma was statistically evaluated by Spearman correlation analysis. Associations miR-150 and/or IGF2BP1 expression with various clinicopathological characteristics of osteosarcoma were analyzed by x^2^ tests. Survival analyses were performed using the Kaplan-Meier and the log-rank tests. *P* < 0.05 was used to indicate a statistically significant difference.

## Results

### Downregulation of miR-150, and Upregulation of IGF2BP1 mRNA and Protein in Human Osteosarcoma Tissues

**Fig. 1:**
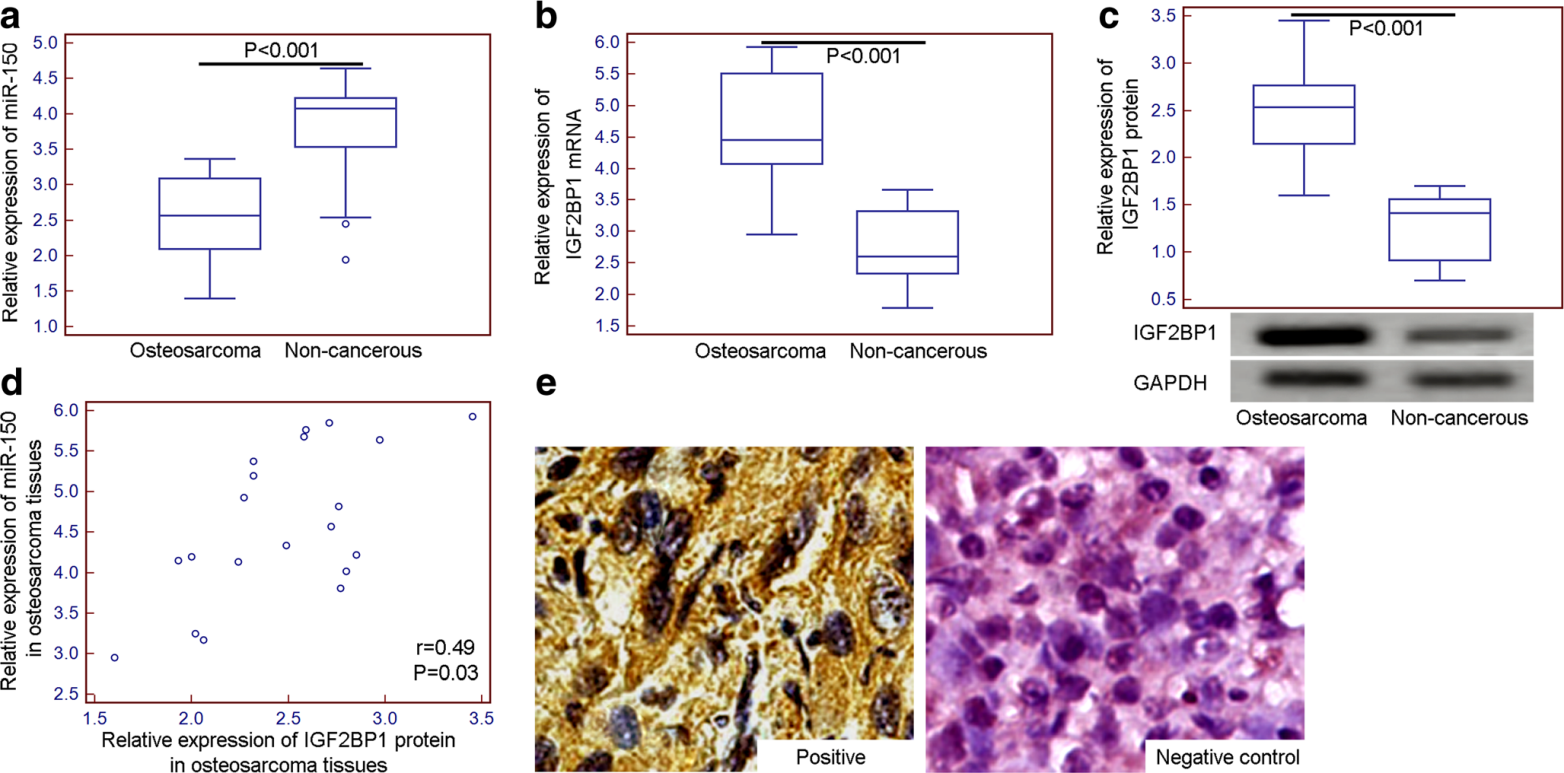
**Downregulation of miR-150, and upregulation of IGF2BP1 mRNA and protein in human osteosarcoma tissues. a** Relative expression of miR-150 in osteosarcoma and matched adjacent non-cancerous tissues; **b** Relative expression of IGF2BP1 mRNA in osteosarcoma and matched adjacent non-cancerous tissues; **c** Relative expression of IGF2BP1 protein in osteosarcoma and matched adjacent non-cancerous tissues; **d** Correlation between miR-150 and IGF2BP1 protein expression in osteosarcoma tissues; **e** Positive immunostaining of IGF2BP1 protein in ostesarcoma tissues and negative control. (×400 magnification)

The expression level of miR-150 in osteosarcoma tissues was significantly lower than that in adjacent noncancerous tissues (tumor vs. normal: 2.55 ± 0.61 vs. 3.83 ± 0.76, *P* < 0.001, Fig. 1a), while the expression levels of IGF2BP1 mRNA and protein were both markedly increased in osteosarcoma tissues (for IGF2BP1 mRNA, tumor vs. normal: 4.60 ± 0.93 vs. 2.75 ± 0.62, *P* < 0.001, Fig. 1b**;** for IGF2BP1 protein, tumor vs. normal: 2.47 ± 0.43 vs. 1.25 ± 0.36, *P* < 0.001, Fig. 1c). Importantly, the expression levels of IGF2BP1 mRNA in osteosarcoma tissues were positively correlated with that of IGF2BP1 protein in osteosarcoma tissues (Spearman correlation *r* = 0.49, *P* = 0.03, Fig. 1d).

According to the observation of immunochemistry, the positive immunostaining of IGF2BP1 protein was predominantly localized in cytoplasm of tumor cells in primary osteosarcoma tissues (Fig. 1e). The median values of miR-150 (2.56) and IGF2BP1 protein IRS (5.62) in all 100 osteosarcoma tissues were respectively used as cutoff points to classify the patients with osteosarcomas into miR-150-low (*n* = 52, 52.00%), miR-150-high (*n* = 48, 48.00%), IGF2BP1-low (*n* = 49, 49.00%) and IGF2BP1-high (*n* = 51, 51.00%) expression groups.

### Associations of miR-150 and/or IGF2BP1 Protein with Various Clinicopathological Features of Human Osteosarcoma

Osteosarcoma patients with miR-150-low expression or IGF2BP1-high expression more frequently had high tumor grade (both *P* = 0.02), positive metastasis (both *P* = 0.001) and recurrence (both *P* = 0.001), and poor response to chemotherapy (both *P* = 0.01). However, there were no associations of miR-150-low expression or IGF2BP1-high expression with other clinicopathological features of patients with osteosarcomas, including age, sex, tumor site and histological type (Table 1).

Among 100 patients with osteosarcomas, 32 (32.00%), 20 (20.00%), 19 (19.00%) and 29 (29.00%) were respectively in miR-150-low/IGF2BP1-high, miR-150-low/IGF2BP1-low, miR-150-high/IGF2BP1-high, miR-150-high/IGF2BP1-low groups. Moreover, the miR-150-low/IGF2BP1-high expression was significantly associated with high tumor grade (*P* = 0.01), positive metastasis (*P* < 0.001) and recurrence (*P* < 0.001), and poor response to chemotherapy (*P* = 0.001) of patients with osteosarcomas (Table 1).

### Associations of miR-150 and/or IGF2BP1 Protein with Patients’ Prognosis in Human Osteosarcoma

**Fig. 2:**
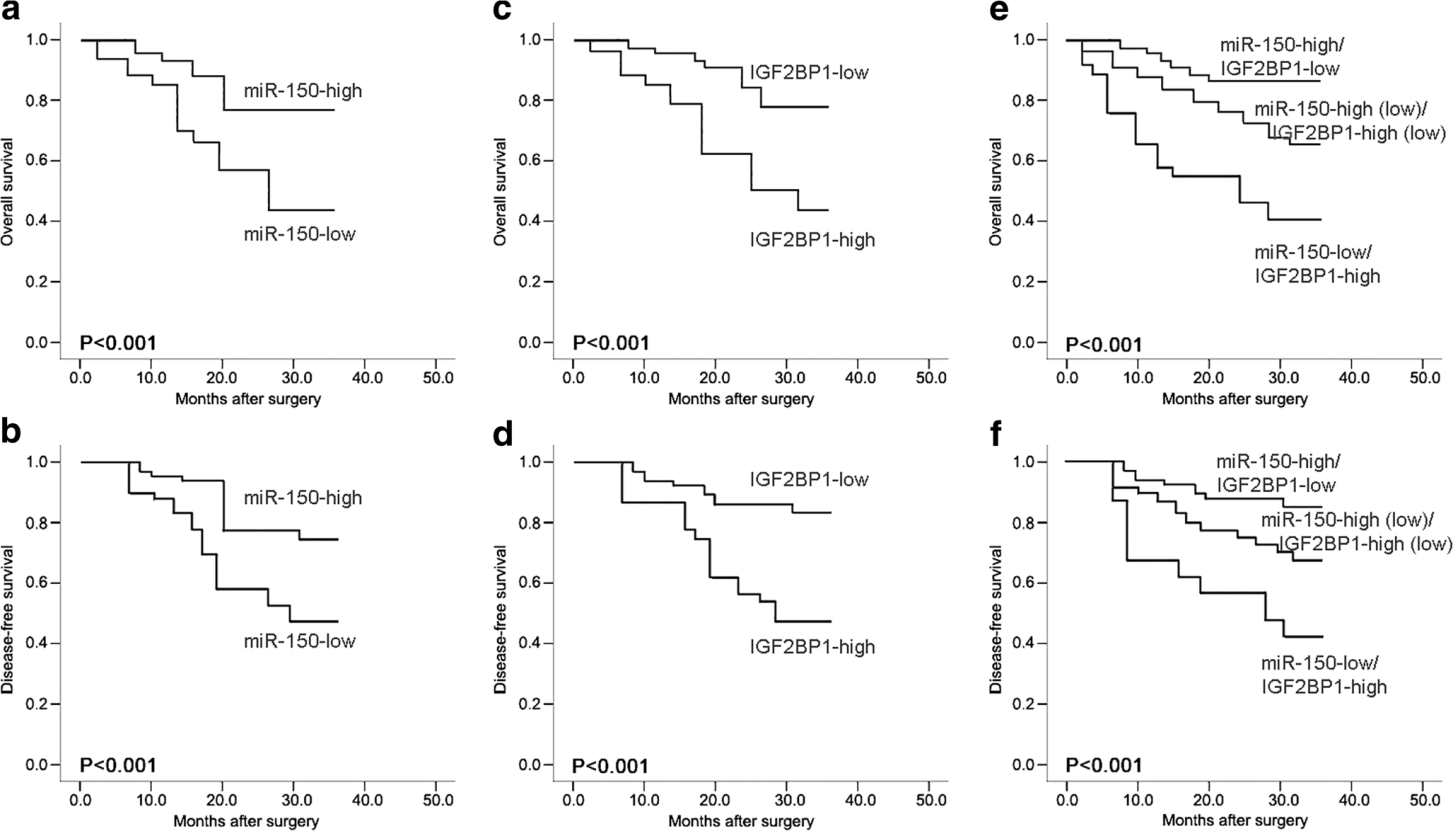
**Kaplan-Meier survival curves for osteosarcoma patients based on miR-150** (**a** for overall survival; **b** for disease-free survival), IGF2BP1 protein (**c** for overall survival; **d** for disease-free survival) and miR-150/IGF2BP1 protein (**c** for overall survival; **f** for disease-free survival) expression

Kaplan–Meier and log-rank tests showed that osteosarcoma patients with miR-150-low or IGF2BP1-high expression had shorter overall and disease-free survivals than those with miR-150-high or IGF2BP1-low expression (all *P* < 0.001, Fig. 2a, b, c and d). More importantly, both overall and disease-free survivals of osteosarcoma patients with miR-150-low/IGF2BP1-high were shortest, compared to miR-150-low/IGF2BP1-low, miR-150-high/IGF2BP1-high, miR-150-high/IGF2BP1-low groups (Fig. 2e, f, respectively; both *P* < 0.001).

Cox proportional hazard model confirmed that miR-150 expression (for overall survival: RR 7.33, 95%CI, 1.10–15.69, *P* = 0.01; for disease-free survival: RR 7.92, 95%CI, 1.12–16.83, *P* = 0.01), IGF2BP1 protein expression (for overall survival: RR 6.62, 95%CI, 0.83–13.02, *P* = 0.02; for disease-free survival: RR 6.86, 95%CI, 0.92–14.09, *P* = 0.02), and miR-150/IGF2BP1 expression (for overall survival: RR 8.52, 95%CI, 1.16–18.36, *P* = 0.001; for disease-free survival: RR 8.89, 95%CI, 1.19–18.98, *P* = 0.001) were all independent prognostic factors of unfavorable survival in human osteosarcoma (Table 2).

**Table 2:** Multivariate survival analysis of overall and disease-free survivals in 100 patients with osteosarcomas

Variables	Overall survival			Disease-free survival		
	Relative risk (RR)	95%CI	P	RR	95%CI	P
Tumor grade	2.42	0.61–5.16	NS	3.37	0.70–7.38	NS
Response to pre-operative chemotherapy	7.28	1.08–15.11	0.01	8.25	1.11–17.99	0.006
Metastasis status	6.44	0.96–13.62	0.02	6.92	1.00–14.06	0.01
Recurrence status	3.26	0.68–7.02	NS	3.59	0.71–7.83	NS
miR-150 expression	7.33	1.10–15.69	0.01	7.92	1.12–16.83	0.01
IGF2BP1 expression	6.62	0.83–13.02	0.02	6.86	0.92–14.09	0.02
miR-150/IGF2BP1 expression	8.52	1.16–18.36	0.001	8.89	1.19–18.98	0.001

## Discussion

Growing evidence shows that osteosarcoma may be caused by the accumulation of genetic and epigenetic alterations. There is an ongoing necessity to identify candidate markers of diagnosis and prognosis, novel therapeutic targets and signaling pathways which are involved into osteosarcoma progression. In the present hospital-based case study, we investigated the clinical relevance of miR-150 and its target gene IGF2BP1 in human osteosarcoma for the first time. On the basis of our data, altered levels of miR-150 and IGF2BP1, alone or in combination, were all significantly associated with advanced clinicopathological features and short survival of patients with osteosarcomas, implying that the imbalance of miR-150-IGF2BP1 axis may lead to aggressive progression and poor prognosis of this malignancy.

A large number of recent studies have revealed that miRNAs may be potential diagnostic and prognostic markers, as well as therapeutic targets for the treatment of various malignant diseases. miR-150, localized on chromosome 19q13, has been identified as a hematopoietic-specific miRNA [21]. In addition to hematological malignancies, miR-150 is implicated in a variety of solid tumors, including glioma [22], osteosarcoma [13–16], esophageal cancer [23], breast cancer [24], non-small cell lung cancer [25], gastric cancer [26], cholangiocarcinoma [27], hepatocellular carcinoma [28], pancreatic cancer [29], colorectal cancer [30], cervical cancer [31], ovarian cancer [32], and prostate cancer [33]. Functionally, miR-150 may act either as an oncogene or a tumor suppressor by regulating various target genes with a cancer type-dependent manner. For example, miR-150 suppressed colorectal cancer cell migration and invasion via targeting MUC4 [30]; miR-150 inhibited hepatoma cell migration and invasion by regulating MMP14 [28]; miR-150 predicted a favorable prognosis in patients with epithelial ovarian cancer, as well as inhibited cell invasion and metastasis by suppressing transcriptional repressor ZEB1 [32]. In contrast, miR-150 over-expression promoted growth, clonogenicity and reduces apoptosis in breast cancer cells through regulating the Pro-Apoptotic Purinergic P2X7 Receptor [24]; miR-150 promoted cellular metastasis in non-small cell lung cancer by targeting FOXO4 [25]. Previous studies [13–15], together with our data here, confirmed that miR-150 was downregulated in osteosarcoma tissues compared to matched adjacent noncancerous tissues. In addition, we found that the decreased expression of miR-150 was significantly associated with high tumor grade, presence of metastasis and recurrence, as well as poor response to chemotherapy. We also revealed a novel role of miR-150 in patients’ prognosis in osteosarcoma.

Together with IGF2BP2 and IGF2BP3, IGF2BP1 belongs to a highly conserved protein family-the IGF2BP protein family, and is characterized by the high expression during the period between zygote and embryo stages [34]. It is emerging as a key regulator of mRNA metabolism with a specific role in controlling the localization, translation or turnover of specific mRNA targets [35]. Accumulating studies observed the abnormal expression of IGF2BP1 in various human cancer types, and indicated its oncogenic roles. For example, depletion of IGF2BP1 inhibited proliferation and induced apoptosis of liver cancer cells in vitro, and suppressed tumor growth of murine xenograft in vivo [36]; IGF2BP1 was commonly up-regulated both in human cervical cancer tissues and cell lines, and was identified as a direct target of a tumor suppressive miRNA-miR-140-5p [37]. Especially, IGF2BP1 mRNA expression was inversely correlated with the level of miR-150 in osteosarcoma tissues, and the downregulation of endogenous IGF2BP1 exhibited similar effects of overexpression of miR-150 in this malignancy [15]. Consistently, our data here confirmed the decreased expression of IGF2BP1 at both mRNA and protein levels and also shown its cytoplasmic localization in malignant cells of osteosarcoma tissues. Moreover, the significant associations of IGF2BP1 overexpression with advanced clinicopathological characteristics and poor prognosis in osteosarcoma patients were also determined. More interestingly, in addition to the clinical implications of the combined miR-150 and IGF2BP1, we also verified that this combination had more significant prognostic value than the two markers alone.

In conclusion, our data suggest that miR-150 and its downstream target IGF2BP1 may be a crucial axis for the development, progression and patients’ prognosis of ostesarcoma. The newly identified miR-150/IGF2BP1 axis might be a novel potential therapeutic target for osteosarcoma treatment. Meanwhile, there are still some limitations need further solving: At first, the follow-up time of the clinical cohort used in this study was relatively short, which may lead to some unexpected results, for example, tumor grade was not identified as a prognostic factor in our survival analysis; Secondly, why miR-150-IGF2BP1 axis was dysregulated in osteosarcoma; Thirdly, whether there are other mechanism for the involvement of miR-150-IGF2BP1 axis in osteosarcoma. Exploration of the above questions may improve our understanding of miR-150-IGF2BP1 axis in human osteosarcoma.
